# Advanced Drug Delivery Platforms for the Treatment of Oral Pathogens

**DOI:** 10.3390/pharmaceutics14112293

**Published:** 2022-10-26

**Authors:** Seyed Ebrahim Alavi, Aun Raza, Max Gholami, Michael Giles, Rayan Al-Sammak, Ali Ibrahim, Hasan Ebrahimi Shahmabadi, Lavanya A. Sharma

**Affiliations:** 1 School of Medicine and Dentistry, Griffith University, Gold Coast, QLD 4215, Australia; 2 School of Pharmacy, Jiangsu University, Zhenjiang 212013, China; 3 Immunology of Infectious Diseases Research Center, Research Institute of Basic Medical Sciences, Rafsanjan University of Medical Sciences, Rafsanjan 7717933777, Iran

**Keywords:** antibacterial agents, drug resistance, infection control, microbiology, oral pathology

## Abstract

The oral cavity is a complex ecosystem accommodating various microorganisms (e.g., bacteria and fungi). Various factors, such as diet change and poor oral hygiene, can change the composition of oral microbiota, resulting in the dysbiosis of the oral micro-environment and the emergence of pathogenic microorganisms, and consequently, oral infectious diseases. Systemic administration is frequently used for drug delivery in the treatment of diseases and is associated with the problems, such as drug resistance and dysbiosis. To overcome these challenges, oral drug delivery systems (DDS) have received considerable attention. In this literature review, the related articles are identified, and their findings, in terms of current therapeutic challenges and the applications of DDSs, especially nanoscopic DDSs, for the treatment of oral infectious diseases are highlighted. DDSs are also discussed in terms of structures and therapeutic agents (e.g., antibiotics, antifungals, antiviral, and ions) that they deliver. In addition, strategies (e.g., theranostics, hydrogel, microparticle, strips/fibers, and pH-sensitive nanoparticles), which can improve the treatment outcome of these diseases, are highlighted.

## 1. Introduction

The oral cavity, as a component of the digestive system, comprises various vital structures, such as teeth, periodontal tissues, and oral mucosa. It is considered a complex ecological niche, including more than 700 microorganisms. This oral microbiota inhibits the growth of pathogenic microorganisms and participates in preserving the stability and balance of oral microecology [1]. Alteration in the composition of oral microbiota, owing to changes in diet, poor oral hygiene, and systemic diseases, could cause the dysbiosis of oral microecology [2] and, consequently, oral infectious diseases (e.g., dental caries, periodontitis, peri-implantitis, and oral candidiasis) [1]. These infectious diseases have a high prevalence, in which dental caries by itself affect 2.5 billion people worldwide [3].

**Figure 1:**
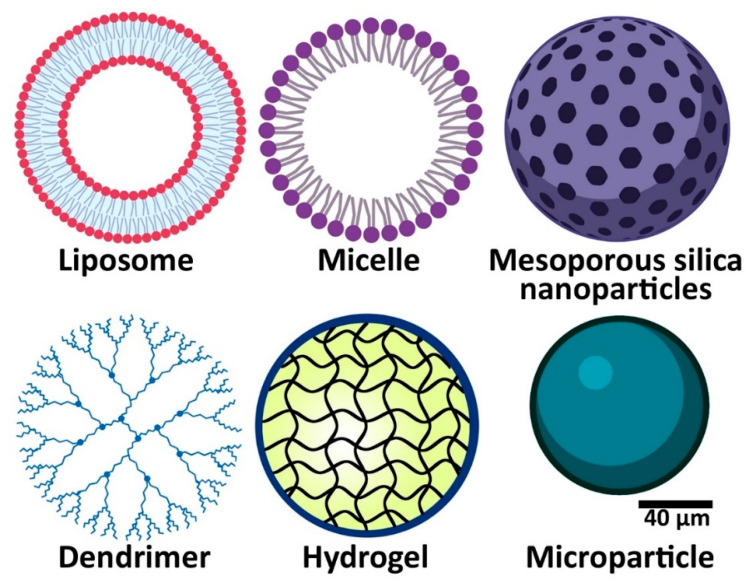
Drug delivery platforms used in the treatment of oral diseases.

Systemic administration has long been used as the most important route for the delivery of therapeutic agents in treating oral diseases; however, it causes complications, such as drug resistance, dysbiosis, and adverse effects, such as impairment in renal and hepatic functions [4]. To overcome these challenges, drug carriers, such as nanoparticles [1,5], hydrogels [6], microparticles [7,8], carbon-based polymers [9], and cyclodextrin-based delivery systems [10] seem to be promising tools as they can (i) control and target drug release, (ii) improve drugs’ pharmacokinetics, and (iii) increase drugs’ bioavailability and selectivity, leading to an improvement in the treatment efficacy [11,12,13,14,15,16,17,18]. In addition, these carriers can improve the administration safety and drug interactions with other body tissues [11]. For example, Chen et al. [19] demonstrated that loading platinum complexes of curcumin into a copolymeric nanoparticle (denoted as Pt-CUR@PSPPN) could decrease the liver toxicity of the therapeutic compounds (Pt-CUR) in A549 xenograft tumor-bearing nude mice, in which the blood concentrations of blood urea nitrogen (BUN) and creatinine in the tumor-bearing mice receiving Pt-CUR@PSPPN, compared to the tumor-bearing mice receiving Pt-CUR, decreased by 16.7 and 4.8%, respectively. Luo et al. [20] synthesized platinum (IV)-prodrug nano-sensitizer and evaluated the acute toxicity of the formulation in female BALB/c nude mice. The results demonstrated that the nanoformulation could increase the maximum tolerated dose (MTD) of cisplatin by 3.3-fold. Fentahun et al. [21] synthesized a thermosensitive hydrogel containing two therapeutic compounds of 5,6-dimethylxanthenone-4-acetic acid (DMXAA) and doxorubicin and evaluated the systemic toxicity of the formulation in a tumor-bearing mouse model (HeLa cells xenograft BALB/c nude mice model) through measuring the weight changes of the animals. The results demonstrated that the hydrogel, containing DMXAA and doxorubicin, compared to DMXAA and doxorubicin, caused a decrease of 2.2% in the body weight loss of the tumor-bearing animals. Among drug carriers, nanoscopic drug delivery systems (DDSs) have received increasing attention for dental applications [11]. Structurally, there are various types of nanoparticles, such as nanospheres, nanofibers, nanocapsules, core-shell, and mesoporous nanoparticles [5,11,22,23,24,25,26,27]. In addition to nanoparticles, other DDSs, such as microparticles and hydrogels, have been used for controlling oral infectious pathogens (Figure 1) [1].

This literature review provides an overview of the application of various DDSs to deliver therapeutic agents for the treatment of oral pathogens. In addition, future directions for their development, with an emphasis on the strategies, such as theranostics and pH-sensitive nanoparticles, hydrogel, microparticles, and strips/fibers, are discussed.

## 2. Drug Delivery Systems for Oral Pathogens Treatment

### 2.1. Nanoparticles

Nanoparticles are the most important carriers of antibacterial drugs for the treatment of oral infectious diseases [1]. Typically, they are copolymer-based nanoparticles, i.e., they are constructed from different polymers, such as poly(lactic-co-glycolic acid) (PLGA) and poly(ethylene glycol)(PEG), and biopolymers, such as lipids, chitosan, and alginate. They benefit from biocompatibility and biodegradability and could be simply modified or combined for drug delivery [1]. Many DDSs, such as protein-polysaccharide coacervate-based nano-carrier systems, are bio-adhesive particles that can adhere to oral mucosa or tooth surface to release a drug locally in a sustained manner [28,29]. For instance, Niaz et al. [29] synthesized nicin-loaded protein-polysaccharide coacervate-based nano-carrier systems (PPC-NCS) using sodium caseinate-sodium alginate. They then evaluated the efficacy of PPC-NCS in adsorbing mucin and determined the profile of nicin release from nicin-loaded PPC-NCS. To evaluate the mucin adsorption efficacy, PPC-NCS solution and mucin type II (1 mg/mL) at the ratio of 1:1 were mixed at room temperature for 1 h. The suspension was then centrifuged (13,000 rpm, 25 min) to obtain the supernatant. The absorbance of the supernatant was then determined at 263 nm, and the amount of free mucin was measured using the standard curve. The amount of adsorbed mucin on the PPC-NCS surface was calculated. Also, the efficacy of nicin-loaded PPC-NCS to control drug release was evaluated in vitro using simulated salivary fluids. The results of mucoadhesive studies on the mucosa of the oral cavity demonstrated that nisin-loaded PPC-NCS, compared to the standard nisin, was 76.7% more potent in adsorbing mucin. In addition, the results of the drug release study demonstrated that the formulation released nisin in a sustained manner, in which ~84% of the loaded drug was released after 32 min at pH 6.8 and 37 °C in simulated salivary fluids [29]. Also, in another study [30], chitosan-based bionanocomposites containing chlorhexidine were prepared, and their profile of drug release and mucoadhesiveness were measured. To evaluate the mucoadhesiveness, mucin (0.1 mg/mL) with and without bionanocomposite formulations (1 mg/mL) was rehydrated in phosphate-buffered saline (PBS, pH 7.4), and a material with an elastic dominant gel like property was prepared. After 18 h incubation at room temperature, a drop of mucin gel was dried by vacuum and gold-coated. The gold-coated gel was then imaged using scanning electron microscopy. Also, the drug release study was performed in vitro at room temperature with the dialysis bag technique. The results demonstrated that chlorhexidine-loaded bionanocomposites had good mucoadhesive properties. In addition, the results showed that the bionanocomposites could release the drug in a prolonged and sustained manner without the initial burst release, in which ~20% of the loaded drug was released after 24 h at pH 6.8.

Nanoparticles can be used to modify dental materials without affecting their basic properties [1]. Also, they could be modified with pH-responsive groups, such as tertiary amine, to structurally respond to an acidic environment and release their cargo [31]. In the oral cavity, sugars are fermented by bacteria within biofilms, resulting in a highly acidic environment, in which the pH values in human dental biofilm, which is also known as plaque, often decrease to 4.5 or even less, especially after contact with cariogenic food products, such as sucrose and starch [31,32]. Thus, the persistent acidic pH (~4.5–5.5) in the plaque at the sites of active caries can occur [31,32]. The niches with low pH promote EPS synthesis, while cariogenic organisms, such as *S. mutans*, continue to multiply, leading to the continuous accumulation of biofilm, the acid-dissolution of tooth enamel and, eventually, the occurrence of dental caries [31]. Akram et al. [33] synthesized pH-sensitive PLGA-modified mesoporous silica nanoparticles (MSN), and the formulation was loaded with chlorhexidine (CHX) and embedded into experimental resin-based dentin adhesives at the weight ratios of 5 and 10 wt%. The formulation released CHX in a pH-dependent manner, in which the highest percentage of drug release, which was ~55% of the loaded drug after 30 days, was observed for 10 wt% CHX-loaded/MSN-PLGA modified adhesive at pH 5, while this value was ~41% at pH 7.4 [33]. Zhang et al. [34], in another study, synthesized a pH-responsive core-shell nano micelle. The resulting formulation, methoxypolyethylene glycol-b-poly-2-(diisopropylamino) ethyl methacrylate (mPEG-b-PDPA), was loaded with bedaquiline, and the profile of drug release from the formulation was evaluated at pH 7, 6, and 5. The results demonstrated that at pH 7, bedaquiline was released slowly in the first 12 h, in which the amount of drug release did not exceed 35% of the loaded drug, and the cumulative amount of the drug release reached over 90% after 8 days. At pH 6, the amount of bedaquiline released in the first 12 h was 70.3% and reached 94.8% on the third day, while at pH 5, the amount of drug released was 92.2% after 3 h [34]. Table 1 demonstrates various drug carriers, including nanoparticles used for the treatment of oral infectious diseases.

**Figure 2:**
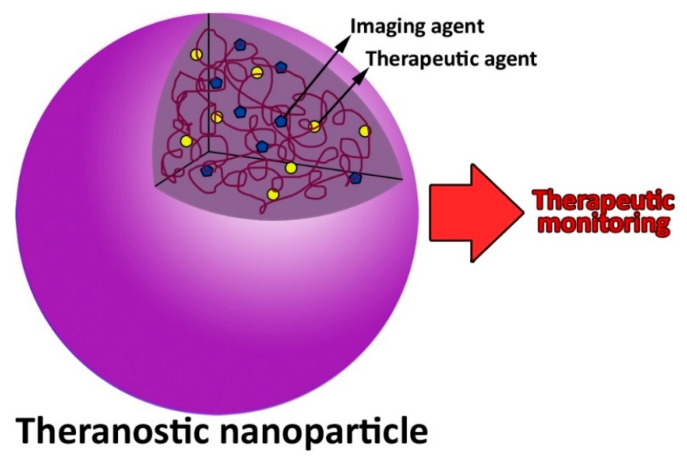
A theranostics nanoparticle used for disease diagnosis, therapy, and monitoring.

Nanoparticles can be also used as theranostic agents (Figure 2). They contain two radioactive drugs, one of which is used to diagnose, and the other is used to treat, resulting in an improvement in the treatment outcomes [44].

### 2.2. Oral Stimuli-Responsive Drug Delivery Systems

The oral cavity, as a unique ecosystem, has several anatomic microniches representing complex physicochemical properties, such as pH, oxygen, temperature, or redox potential [45]. In the healthy state, these features (i.e., pH (6.2–7.6 [46]), oxygen, temperature (37 °C [47]), and redox potential) are in a stable state; however, when infectious diseases occur, there would be a disturbance in the stability, and these properties change [47].

Oral stimuli-responsive DDSs, also known as smart DDSs, have been recently investigated [48,49]. These DDSs could react to the changes in the physicochemical properties of the oral cavity and cause more accurate drug delivery or release. In oral medicine, two types of stimuli-responsive DDSs have been investigated, nano and injectable DDSs [50]. Among the nano DDs, pH-responsive DDSs are the most commonly used carrier in the oral cavity [50]. In pH-responsive DDSs, the carriers are decorated with pH-sensitive groups, such as amines or acid-labile bonds, and the drug release is controlled by protonation/deprotonation reaction or breakage of the chemical bonds during pH change [50].

Among injectable DDSs, hydrogels are more predominant and are developed using a Sol-Gel method [1]. This type of DDSs could also be modified to respond to the changes in the physicochemical properties of the oral cavity, such as pH and temperature [1]. For instance, in one study, Chang et al. [51] synthesized a naringin-loaded thermo-gelling and pH-responsive carrier that could respond to temperature and pH changes. The hydrogel was synthesized from carboxymethyl-hexanoyl chitosan, β-glycerol phosphate, and glycerol that was constantly fluidic at 4 ℃, while the synthesized hydrogel became gel rapidly at 37 °C. The hydrogel could also release naringin faster at pH 5.5 due to the protonation of amine groups at acidic pH [51]. Overall, oral stimuli-responsive DDSs lead to controlled and targeted drug release and, consequently, an enhancement in therapeutic efficacy a reduction in the side effects of the drugs.

### 2.3. Hydrogels

Hydrogels are water-soluble polymeric structures with high porosity. They are three-dimensional, cross-linked networks that could readily swell in an aqueous environment and absorb water or biological fluid and, as a result, form a gel matrix [6]. Hydrophilic groups (e.g., -OH, -CONH-, CONH_2_-) are responsible for hydrogel formation [52]. Hydrogels are well-biocompatible and are similar to the native extracellular matrix owing to their high water content [53]. This DDS can load a large amount of drug due to its porous structure and release the drug in a controlled manner due to its capability of swelling in an aqueous environment. In one study, Tarawneh et al. [54] developed a chlorhexidine-loaded cellulose-based hydrogel and measured the drug loading efficiency and the hydrogel efficacy to control the drug release. The results demonstrated that the hydrogel was synthesized with a drug loading efficiency of 58% and could slowly release the drug, in which less than 10% of the loaded drug was released within 96 h [54]. In another study, ref. [55] fluconazole-loaded hyaluronic acid-based hydrogel was synthesized and characterized in terms of drug loading efficiency and profile of drug release. The results demonstrated that the hydrogel was synthesized with the drug encapsulation efficiency of 83.3% that could preserve ~15% of the loaded drug after 3 h, and released the remaining loaded drug (~85%) in a controlled manner [55]. Most hydrogels have exceptional bioadhesivity and could adhere to the tooth surface and oral mucosa for sustained drug release [53]. Kong et al. [41] developed a histatin-5 bioadhesive hydrogel using hydroxypropyl methylcellulose. The results of viscosity measurement and drug release studies demonstrated that hydrogel viscosity decreased by increasing the temperature, in which the viscosity was 317.84 ± 6.92 centipoise (cP) at room temperature, while this value was equal to 197.73 ± 0.76 cP at 37 °C. Also, the results of the drug release study demonstrated that histatin-5 was released from the formulation in a controlled, sustained manner, in which ~98% of the loaded drug was released after 2 h at 37 °C. Hydrogel DDSs have been widely used for the treatment of periodontitis and peri-implantitis (Table 1) [1]. They can be readily administered in liquid form at the site of drug absorption, where they swell and form a stable gel, which could prolong the residence time of the active substance [1]. The drug, in the liquid dosage form, is delivered to the periodontal tissue, and after reaching the target site, the liquid dosage form changes into a gel dosage form through a sol-gel transition process, and the drug is then released in a sustained manner [1,56,57]. In one study, Garala et al. [56] synthesized an in situ hydrogel containing chlorhexidine hydrochloride using a cold method and different polymers, such as poloxamer 188, poloxamer 407, gellan gum, and carbopol 934P. The results demonstrated that only hydrogels prepared from 15% *w*/*v* and 20% *w*/*v* concentration of poloxamer 407 could form hydrogels at various ranges of body temperature (28–39 °C).

### 2.4. Microparticles

Polymer-based microparticles have been studied to improve the therapeutic effects of antimicrobial drugs against oral infectious agents [7,8]. Various synthetic (e.g., PLGA) and natural (e.g., chitosan) polymers have been utilized in the construction of microparticles for drug delivery [58]. Kawatika et al. [59], in one study, demonstrated that chitosan microparticles could decrease the bacterial (*Streptococcus mutans* (*S. mutans*)) viability (6.5 × 10^3^ versus 2 × 10^5^ colony-forming unit (CFU)/cm^2^) and increase the acidogenicity (pH 7.3 versus 5) more than chitosan aqueous dispersion; therefore, the results indicated that chitosan microparticles were more potent by ~30.8-fold in inhibiting the growth of mature biofilms. The results of another study [60] demonstrated that doxycycline-loaded PLGA microspheres released doxycycline in a sustained manner in the periodontal pocket of patients with chronic periodontitis. Currently, a drug-loaded microparticle (Arestin®), which is prepared from bioresorbable polymer, poly (glycolide-co-DL-lactide) and minocycline hydrochloride antibiotic, is available in the market to treat patients with adult periodontitis [58].

### 2.5. Strips/Fibers

Strips and fibers, with a polymeric matrix, have been utilized for antimicrobials (e.g., chlorhexidine, doxycycline, tetracycline, minocycline, and metronidazole) delivery as an accessory treatment in periodontology [58]. They can be constructed in the proper dimension to be inserted into the periodontal pocket to achieve desired clinical results [61]. Strips and fibers are appropriate DDSs for the treatment of periodontitis owing to their (i) biocompatibility and biodegradability, (ii) ability to completely fill the pockets, and (iii) great mucoadhesion features, which lead to their strong retention capability on the target site. Local drug delivery into the periodontal pocket causes the target sites to become directly accessible. It also improves patient compliance and resolves gastrointestinal problems resulting from oral drug delivery. In addition, local drug delivery into the periodontal pocket is a noninvasive, pain-free, and easy-to-use that avoids the hepatic first-pass metabolism, improves the drugs’ therapeutic efficacy, and reduces the treatment cost. Moreover, this route prolongs the drug action and can be considered a reliable drug delivery route in patients who cannot swallow [61]. Strips and fibers can be synthesized from various polymers and their combinations. Acrylic polymers have been extensively used to synthesize strips and fibers [4]; however, owing to their adverse effects, such as non-absorbability and removal from the body that is required after therapy, other polymers, such as cellulose derivatives (hydroxypropyl cellulose, hydroxypropyl methyl- cellulose, ethyl cellulose), polycaprolactone, polyhydroxy-butyric acid, poly- methylmethacrylate, and PLGA, have been used for their synthesis [58]. PerioChip is a commercial strip composed of a biodegradable matrix of hydrolyzed gelatin containing chlorhexidine gluconate. PerioChip can preserve the drug concentration above the minimum inhibitory concentration (MIC) for > 99% of periodontal pocket flora for up to 9 days [62]. Also, Actisite^®^ is a commercial periodontal fiber for use in the periodontal pocket. It is composed of a monofilament of ethylene/vinyl acetate copolymer and tetracycline hydrochloride antibiotic that releases the antibiotic continuously for 10 days [63].

## 3. Drugs Used in the Treatment of Oral Pathogens

### 3.1. Antibiotic Delivery

**Figure 3:**
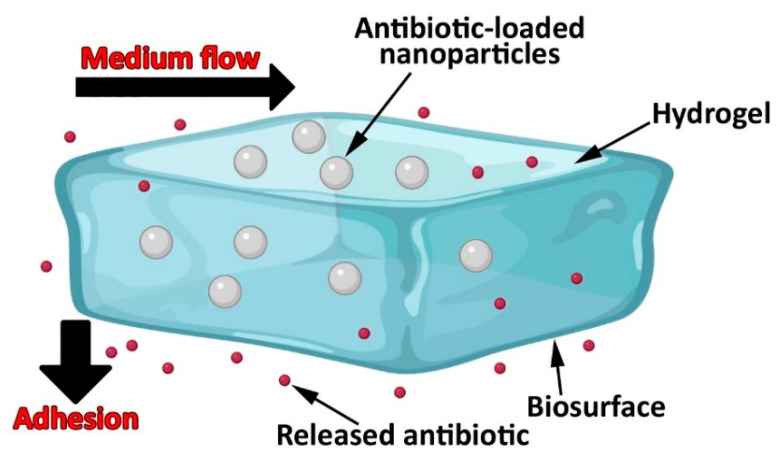
Localized antibiotic delivery using an adhesive nanoparticle-hydrogel hybrid system to prevent the growth of bacteria under flow conditions. In this platform, dopamine methacrylamide, comprising a functional catechol group, is used as an adhesive moiety.

Bacterial biofilms cause a significant challenge in oral health. The biofilms are produced when bacteria cluster together, producing extracellular polymeric substances (EPSs) around each bacterial cell. The EPSs contain various proteins and polysaccharides, that produce binding sites. The microenvironment of EPSs allows bacteria to adhere to each other and stick to biological and non-biological surfaces, resulting in drug resistance. In addition, some bacteria (e.g., *S. mutans* and lactobacilli) can develop an acidic environment, which decreases the effectiveness of the antibiotics [11]. Moreover, the shear forces in the oral cavity, due to the action of the tongue and oral mucosa, further increase the treatment challenges of bacterial infections. To overcome these challenges, new platforms of DDSs can be utilized to improve antibiotic-pathogen interactions and modulate drug dosage [11]. In this regard, a new strategy that exploits shear forces in the oral cavity has been investigated by Zhang et al. [64]. Figure 3 demonstrates a nanoparticle-hydrogel hybrid system that could release antibiotics effectively under shear forces. The results of Zhang et al. [64] study demonstrated that the bio-adhesive nanoparticle-hydrogel delivery system could release the loaded drug locally in a controlled manner, enabling controlled and sustained drug release kinetics. To evaluate the drug release from the nanoparticle-hydrogel delivery system, 0.5 mL of the formulation was transferred into a dialyzer, and a 100 nm pore-size dialysis membrane was used. The dialyzer was immersed in 100 mL of PBS and stirred (37 °C). At various time points, 1 mL of the solution was replaced with 1 mL of the fresh PBS. The drug concentration in the collected samples was calculated. The results also demonstrated that this system had excellent adhesion and antibiotic retention in biological environments (e.g., bacterial film, a mammalian cell monolayer, and mouse skin tissue) under high-shear stress conditions. In addition, this bioadhesive nanoparticle-hydrogel system was found potent in treating other diseases by selecting the proper drug and nanoparticle cargo. The adhesion and viscoelasticity features of the nanoparticle-hydrogel delivery system could be adjusted to meet the need for shear stresses under peculiar physiological conditions. This system was also found to be completely safe and nontoxic as it did not cause any seeable skin reaction or toxicity during the 7-day treatment process. However, it may be necessary to match the duration of gel adhesion with the time span for the drug release as the gel without antibiotics could be potentially susceptible to bacterial colonization. This issue can be addressed by either regulating the hydrogel degradation rate or regulating the rate of antibiotic release from nanoparticles. Overall, this bio-adhesive nanoparticle-hydrogel delivery system that utilizes adhesive force to overcome high shear forces demonstrated a high potential for long-lasting, safe, and efficient localized delivery of different therapeutics [64].

### 3.2. Antifungal Delivery

Among oral fungal infections (e.g., candidiasis, histoplasmosis, aspergillosis, and coccidioidomycosis), candidiasis, caused by *Candida albicans* (*C. albicans*), is the most common infection that can develop hyperplastic candidiasis, leading to carcinoma [11,65]. There are some hypothetical molecular mechanisms by which *C. albicans* can cause dysplasia and malignant neoformations. These mechanisms include (i) the generation of endogenous nitrosamines, (ii) the overexpression of the transcription factors of P53, Ki-67, and COX-2, (iii) an increase in the generation of acid aspartyl proteinase, (iv) an increase in the production of proinflammatory cytokines, such as interleukin (IL)-1α, IL-1β, IL-6, IL-8, IL-18, and tumor necrosis factor (TNF)-α, (v) an increase in the generation of acetyl-CoA synthetase, (vi) a decrease in the production of β-defensins and an increase in the production of alcohol dehydrogenase enzyme, and (vii) the production of candidalysin as a toxic protein [66]. Fluconazole is an antifungal drug used for the site-specific treatment of *C. albicans* infections [11]. However, the clinical application of fluconazole is associated with side effects, such as headache, liver disease, and the risk of drug resistance. Thus, it is required to use an appropriate DDS to overcome these problems [67]. For this purpose, mucoadhesive nanoparticles have been developed for fluconazole delivery [11]. These particles can be coated with chitosan as a biocompatible and non-toxic compound with antifungal and mucoadhesive properties to improve their efficacy [11]. It has been demonstrated that the use of chitosan, as a surface coating agent, can increase the bioavailability of nanoparticles due to its mucoadhesive property. Mucoadhesive delivery systems can enhance the residence time of the dosage forms at the site of delivery that may result in enhanced bioavailability [68]. In addition to chitosan nanoparticles, other DDSs, such as lipid-based nanoparticles [69], hydrogel [70], and nanofiber [71], have also been used for the treatment of oral candidiasis (Figure 3). Mendes et al. [69] synthesized a miconazole-loaded nanostructured lipid carriers (NLC) dispersion and evaluated its antifungal activity against *C. albicans*. The results demonstrated that the nanoparticles could increase the antifungal activity of the drug by 4-fold. Martin et al. [70] synthesized sodium alginate-based hydrogel containing nystatin and evaluated its antifungal activity against *C. albicans* on pig animals. To evaluate the antifungal activity of the nystatin-loaded hydrogel in vivo, adequate amounts of hydrogel equivalent to 30 mg nystatin was applied on the buccal mucosa of one of the cheeks (9 cm^2^ each one) using a paintbrush. The hydrogel was administrated 4 times a day for 2 days. According to the results, the drug-loaded hydrogel, compared to the standard drug, caused less antifungal activity by 1.7-fold in vitro; however, the results of in vivo study demonstrated that the resulting amounts of nystatin retained in the porcine mucosae of pigs receiving nystatin-loaded hydrogel was 3.38 ± 0.25 µg/g tissue/cm^2^, while there was no nystatin in the porcine mucosae of pigs receiving nystatin solution. Tonglairoum et al. [71], in another study, evaluated the antifungal activity of a clotrimazole-loaded microemulsion-containing nanofiber mats compared to the standard clotrimazole. The results demonstrated that the nanofiber formulation of clotrimazole, compared to the standard clotrimazole, was ~11.4-fold more potent in killing *C. albicans* after 30 min treatment [71].

**Figure 4:**
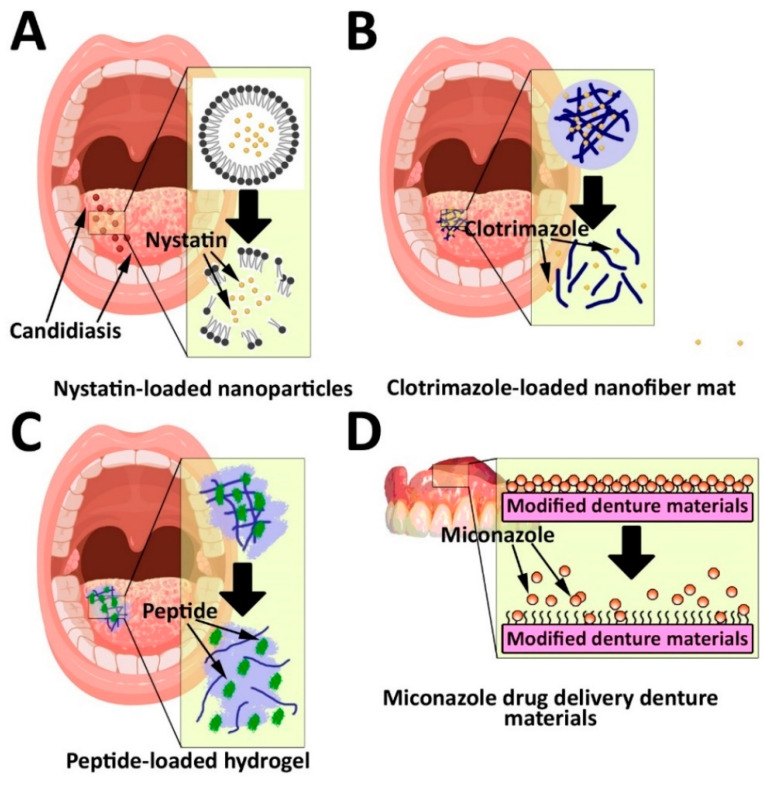
Drug delivery systems for the treatment of oral candidiasis using (**A**) lipid-based nanoparticles, (**B**) nanofiber mat, (**C**) bioadhesive hydrogel, and (**D**) denture materials modified by prolonged miconazole release. The denture materials modified by copolymer improve binding with miconazole, resulting in prolonged drug release.

In addition, various pH-sensitive, polymer-based microparticles with mucoadhesivity have been developed for encapsulating miconazole nitrate. These microparticles can release the drug in a sustained manner (Figure 4D), and improve the miconazole nitrate dissolution rate [11,72,73,74]. In one study, Tejada et al. [74] synthesized miconazole nitrate-loaded microparticles using hydroxypropyl methylcellulose and evaluated the efficacy of the drug-loaded microparticles in controlling the drug release and improving its dissolution rate. The results demonstrated that the particles could control the drug release for 30 min, in which the whole amount of the drug was released from the microparticles after 30 min. Also, the microparticles could improve the dissolution rate of the drug by ~2.7-fold [1].

### 3.3. Antiviral Delivery

Most antiviral drugs, such as atazanavir [75] and erythromycin [76], can specifically target viruses. By now, antiviral drugs are most commonly administered orally, meaning that in addition to the lung, the major proportion of the drugs is distributed to the systemic circulation [77]. Viral infections, such as oral herpes infection, are able to engage the skin of the mouth and oral mucosa. Oral herpes, due to human herpes simplex virus 1 (HHV-1), can cause pain in the lips, tongue, and mouth roof [78], while HHV-2 causes genital herpes. HHV-3 is another human herpes simplex virus that causes chickenpox and herpes zoster. Also, HHV-4 (Epstein- Barr) and HSV-5 (cytomegalovirus) viruses are other viral infection agents that cause infectious mononucleosis. HHV-6 and HHV-7 can cause roseola, which is a viral infection associated with high fever and a skin rash in small children. HHV-8 virus in patients with acquired immunity deficiency syndrome (AIDS) causes Kaposi’s sarcoma [79]. There are a few strategies for the local administration of antiviral drugs. For example, using an antiviral cream for the treatment of labial herpes is the only local route for the treatment of the disease [4]. Nanoparticle application for the delivery of antiviral drugs can be exciting for researchers [80] because they are able to regulate their release kinetics, enhance their bioavailability, control the rate of their dissolution, decrease their side effects, and decrease the treatment cost [11]. In this regard, various types of nanoparticles, such as graphene oxides (GOs) [81], carbon dots (C-dots) [82], and fullerenes [9], have been used for the treatment of viral diseases that will be discussed in more detail.

#### 3.3.1. Carbon-Based Polymers

Carbon-based nanomaterials, such as graphene, carbon dots (C-dots), and fullerenes, have been confirmed for delivering antiviral drugs as they have low cytotoxicity and can be easily manipulated to specify their function. For instance, graphene can directly interact with viruses, while fullerene has inhibitory effects on viral activity [9]. The sharp edges of graphene oxides (GOs) can nullify the virus before its interaction with cells. Moreover, the negative charge of GOs enables them to interact with viruses electrostatically and, as a result, causes an improvement in their antiviral activity. Moreover, graphene oxide can perform photocatalysis, resulting in viruses being photodegraded directly. This further improves the degradation caused by the sharp edges. Graphene oxide nanoparticle systems supported by silver have also shown an increase in their efficacy in degrading viruses. This results from the ability of silver to increase binding to the glycoproteins on the viral membrane and prevents the invasion of viruses into cells [10]. Du et al. [83] synthesized silver nanoparticles modified GOs nanocomposites (GO-AgNPs) and evaluated the antiviral efficacy of the formulation. The results demonstrated that GO-AgNPs could suppress the porcine reproductive and respiratory syndrome virus (PRRSV) infection and inhibit the virus entry into the host cells by an inhibition efficiency of 59.2% [83].

C-dots nanomaterials, a carbon-based nanomaterial, could cause antiviral effects by themselves. Barras et al. [84] synthesized C-dots from 4-aminophenylboronic acid hydrochloride and evaluated their antiviral activity against HSV-1 infection. The results demonstrated that the formulation could inhibit HSV-1 infection in ng/mL concentration range with the half maximal effective concentration (EC_50_) of 80 and 145 ng/mL on Vero and A549 cells, respectively. Du et al. [85], in another study, evaluated the cell toxicity and antiviral effects of C-dots on the replication of pseudorabies virus (PRV) and PRRSV. The results demonstrated that the C-dots had low cytotoxicity against Monkey kidney MARC-145 and Porcine kidney PK-15 cells (cell viability of ˃90% at 0.125 mg/mL of C-dots after 12, 24, 36, and 48 h incubation). Also, C-dots, compared to the control group, could inhibit the PRV entry into the host cells by ~13.2% after 24 h incubation, while this value for PRRSV was ~19.4% [85]. Fahmi et al. [86] synthesized anhydrous citric acid-based C-dots nanoparticles and modified them with carboxyl phenylboronic acid (CBBA; CBBA-Cdots). The toxicity and antiviral effects of C-dots and CBBA-Cdots against MOLT-4 human leukemia cells and human immunodeficiency virus -1 (HIV-1) were then evaluated. The results demonstrated that C-dots and CBBA-Cdots with the size of 2.8 and 6.2 nm, respectively, were synthesized. Both nanoparticles were nontoxic towards MOLT-4 cells (the half of cytotoxic concentration (CC_50_) values of 2901.2 and 1991.9 μg/mL for C-dots and CBBA-Cdots, respectively). Also, the results demonstrated that C-dots and CBBA-Cdots could inhibit virus entry into the host cells by half concentration of inhibition (IC_50_) values of 9506.3 and 26.7 μg/mL, respectively. This primarily resulted from the cooperation between hydroxyl and carboxyl surface groups as well as boronic acid to prevent the formation of hydrogen bonding between the viruses and cells [9].

In the case of fullerene, this specific type of carbon-based material has antiviral activity against HIV due to its potency to block the encoded enzymes by preventing the active sites of HIV protease. Generally, fullerenes have low cytotoxicity against human lymphoblastic CEM cells [87]; however, they have low solubility. To address this issue, they can be combined with highly soluble materials, such as alkali metal salts. Fullerenes can produce singlet oxygen particles, which cause the photodynamic inactivation of viruses. Carbon-60-based materials have photosensitizer activity. As photosensitizer activity causes an increase in their cytotoxicity, the decreased solubility perceived in fullerenes and related materials is an advantage owing to the more readily removal of carbon-60-based materials from the body. In general, fullerenes have demonstrated promising efficacy in treating several viral diseases, such as hepatitis C virus (HCV), respiratory syncytial virus (RSV), H1N1, herpes simplex virus, human cytomegalovirus, Zika, and Dengue viruses [9].

#### 3.3.2. Cyclodextrin-Based Delivery Systems

Cyclodextrins (CDs)-based delivery systems are able to deliver drugs site-specifically at an associated spread rate; thus, they are promising carriers for the treatment of viral infections. They could form hydrophobic inclusion complexes in both solution and solid states, allowing them to change their physical properties easily. Also, CDs-based delivery systems can be chemically modified to improve their properties. For example, 𝛽-CD, as the standard form of CDs, contains 21 hydroxyl groups (seven primary and fourteen secondary hydroxyls), which can be modified with various functional groups [10]. Due to bioadaptability and multifunctional features, CDs are able to reduce the undesirable features of drugs through the formation of inclusion complexes [88].

There are various types of release patterns for CDs as DDSs, including immediate, prolonged, modified, and delayed releases. Immediate release is especially applicable when the effects of an injected drug are needed urgently. In the immediate release, CDs increase the dissolution rate of a drug, which is not very water-soluble. The CDs, such as hydroxypropyl-β-cyclodextrin (HP-β-CD), heptakis-[2,6-di-O-methyl]-β-cyclodextrin (DM-β-CD), sulfobutylether-β-cyclodextrins (SB-β-CDs) and branched 𝛽-CDs, that release the loaded drugs immediately, are generally used for the delivery of low water-soluble drugs while CDs, such as ethylated and acylated 𝛽-CDs, that release the loaded drugs in a prolonged manner are used for the delivery of water-soluble, high dose drugs. Prolonged-release formulations, compared to immediate-release formulations, cause a reduction in the frequency of administering doses to patients as the release of a larger single dose takes a longer time [88]. CDs formulations, such as cyclodextrins in combination with polyoxy 60 hydrogenated caster oil derivative (HCO-60) or in combination with hydroxypropyl cellulose, that cause drug release in a modified manner can be developed from the combination of CD complexes with different carriers [88]. HCO-60 causes an inhibition in the drugs’ crystal formation, while hydroxypropyl cellulose causes a relatively steady rate of drugs’ dissolution within the body. CD-based formulations with modified-release profiles can release the encapsulated drugs continuously over a period of time. These formulations are used for drugs which have poor oral bioavailability and reduced solubility owing to crystal formation. In addition, CDs formulations that release the loaded drug in a delayed manner are used for the delivery of drugs into a specific part of the body by measuring the time needed for a drug to reach the target site and be metabolized [10]. In one study, Donalisio et al. [89] prepared a complex of acyclovir and sulfobutyl ether-β-cyclodextrin and then incorporated the resulting formulation into chitosan nanodroplets. The antiviral efficacy of the formulation was then evaluated using a plaque reduction assay. The results demonstrated that the acyclovir-loaded nanodroplets, compared to acyclovir, were more efficient in inhibiting HSV-2 virus infection by ~2.8-fold [89].

### 3.4. Ion Delivery

*S. mutans* and *S. sobrinus* bacteria ferment carbohydrates and produce organic acids. The intensified acidity promotes the calcium and phosphate ions released from enamel and mineralized dentin. This demineralization process is neutralized by the saliva activity, in which the bicarbonate ions in saliva function as a buffer and cause restoring the normal pH in the oral environment. Also, mineral ions in saliva restore the calcium and phosphate ions on the tooth surfaces (remineralization). Shifting this dynamic physiological balance to demineralization can cause dental caries and, as a result, enamel dissolution [11,90,91]. The delivery of calcium, phosphate, and fluoride ions to block demineralization in the oral environment is a serious challenge for dental researchers for over a century. Herein, the delivery of various ions for dental application will be discussed in more detail.

#### 3.4.1. Fluoride Delivery

Fluoride suppresses the growth of bacteria, which cause dental caries and further acidification of the oral milieu [11,92]. Increasing the concentration of fluoride ions in the saliva causes a decrease in hard tissue demineralization [93]. Fluoride ions, through reacting with hydroxyapatite (HA), incorporate into the HA lattice structure to generate fluorapatite with higher acid-resistance properties [11,92]. HA is a biocompatible agent and has been used in different formulations as a biomimetic compound to prevent enamel caries progression [94] and to relieve dentin hypersensitivity [95]. Also, fluoride ions disrupt the metabolism of bacteria which produce organic acid and suppress caries progression [11,92]. It has been shown that daily consumption of 200 ppm of fluoride suppresses dental caries [96]. Designing novel ion delivery systems to preserve the fluoride ions concentration in the saliva has been reported [97,98,99].

The large surface-to-volume ratio of microparticles and nanoparticles, as fluoride ions delivery systems, enables them to increase their ions loading capacity. These particles can also release fluoride ions in a controlled manner, which causes maintenance of the optimized concentration of the ions and their protective effects for a longer time. For this purpose, nanoparticles, compared to microparticles, are more efficient carriers due to their higher surface-to-volume ratio. For example, calcium fluoride nanoparticle (nano-CaF_2_), compared to traditional glass ionomer cements, causes an increase in the cumulative fluoride release because nano-CaF_2_, compared to traditional glass ionomer cements, has a 20-fold higher surface area [100]. Different ion delivery systems have been synthesized, and their efficacy in delivering fluoride was evaluated [96,97,98,99,101]. Ghafar et al. [96] synthesized thiolated chitosan-based bioadhesive film, the film was loaded with calcium fluoride nanoparticles and lignocaine, and the efficacy of the resulting bioadhesive films in controlling the release of fluoride ions was evaluated. The results demonstrated that the films caused a prolonged release of fluoride ions for 8 h, in which ~94% of the loaded fluoride was released after 8 h at pH 6.8. Keegan et al. [97] synthesized (sodium fluoride) NaF/chitosan microparticles using glutaraldehyde as a cross-linker. The resulting formulation caused the continuous release of fluoride ions up to 6 h at pH 4 and 7, in which ~74 and ~78% of the loaded fluoride were released at pH 4 and 7, respectively. De Francisco et al. [98], in another study, synthesized sodium fluoride-loaded ethylcellulose and sodium fluoride-loaded gelatin microparticles and evaluated the efficacy of the particles in releasing the loaded sodium fluoride in vitro. The results demonstrated that the formulations could release the loaded sodium fluoride in a sustained manner over 8 h, in which ~11.5 and ~60% of the loaded sodium fluoride were released from ethylcellulose microparticles and gelatin microparticles, respectively, after 8 h. In addition, Nguyen et al. [99] synthesized chitosan/fluoride nanoparticles using sodium tripolyphosphate, as a cross-linking agent, and demonstrated that the fluoride ions were released from the particles in a sustained manner at both pH 5 and 7, in which ~58 and ~46.7% of the loaded fluoride were released at pH 5 and 7, respectively, after 24 h. The acidic pH (pH 5) caused an increase in the release of fluoride from the nanoparticles. The results of Nguyen et al. study [99] suggest that these nanoparticles can release fluoride ions in acidic pH and promote hard tissue remineralization [96]. Samarehfekri et al. [101], in another study, synthesized polylactic acid (PLA) nanoscaffold nanomicelles containing NaF and evaluated the efficacy of the nanomicelles in controlling the release of fluoride. The results demonstrated that the nanomicelles released 59% of the loaded fluoride after 4 h at pH 7.4 and 37 °C.

#### 3.4.2. Calcium and Phosphate Delivery

Calcium ions constitute 99% of bone tissue, and the administration of calcium compounds, such as calcium carbonate, calcium lactate, or calcium gluconate, causes the inhibition of osteoporosis and bone loss. Carbonated apatite, with 10 calcium ions and 6 phosphate ions, constitutes the bulk of minerals in enamel [102,103].

Using ion delivery systems, such as HA [104], tricalcium phosphate [11,105], and amorphous calcium phosphate (ACP), for the delivery of calcium and phosphate ions is a promising approach to preventing dental caries by increasing the saturation of these ions in the oral environment [11,106]. Polyamidoamine (PAMAM) dendrimers are hydrophilic polymers with a core of ethylenediamine and amidoamine branching structure that make them capable of absorbing calcium molecules [107]. Liang et al. [103], in a study, prepared PAMAM dendrimers containing calcium and phosphate ions and evaluated their efficacy in preventing tooth decay. The resulting formulation could release calcium and phosphate ions at low pH and neutralize the acidic environment and inhibit dental caries [103]. ACP nanoparticles are not sufficiently stable in the oral environment and are easily converted into a crystalline form, resulting in a decrease in the bioavailability of calcium and phosphate ions for tooth enamel remineralization [11]. To address this issue, Luo et al. [108] used polyacrylic acid to improve ACP stability. Polyacrylic acid-ACP was then loaded into MSNs through electrostatic interaction. The resulting formulation caused the release of calcium and phosphate ions in a sustained manner to remineralize collagen fibrils in demineralized dentin [108]. Also, casein phosphopeptide (CPP) as a cluster protein has been used to enhance the ACP stability and, as a result, to improve the calcium and phosphate ions bioavailability. Mendes et al. [109] demonstrated that CPP-ACP could reduce tooth decay by releasing calcium and phosphate ions into the oral environment.

## 4. Conclusions and Outlook

One of the most important strategies to treat oral infectious diseases is the systemic administration of antimicrobial drugs, where the drugs are distributed throughout the body, resulting in an increase in their side effects and a decrease in the therapeutic effects. Systemic administration can develop drug resistance, dysbiosis, and impairment in renal and hepatic functions. To solve these issues, DDSs are considered an appropriate strategy in order to improve the therapeutic effects of drugs. Using DDSs can lead to a controlled and targeted drug release pattern and improve the drug pharmacokinetics, bioavailability, and selectivity, resulting in an improvement in the treatment outcome. By now, various DDSs, such as nanoparticles (e.g., liposome, chitosan, MSNs, micelle, PLGA, dendrimer), hydrogels (e.g., monomethoxy poly(ethylene glycol)-block-poly(d,l-lactide) (mPEG-PDLLA), hydroxypropyl methylcellulose (HPMC)), microparticles (e.g., poly (glycolide-co-DL-lactide)), and strips/fibers (e.g., biodegradable matrix of hydrolyzed gelatin) have been evaluated and have demonstrated promising results. Investigations have shown that (i) using nanoparticles could release the loaded drugs/agents in a pH-dependent manner to achieve targeted drug delivery, enhance the antibacterial properties of the restorative materials, and improve the antibacterial and antifungal activity of the loaded drugs/agents, (ii) using hydrogels could enhance the half-life of the loaded antibiotics and improve the antifungal and antibacterial activity of the drugs/agents, (iii) using microparticles could enhance the antibacterial activity of the drugs/agents, and (iv) strips/fibers have excellent mucoadhesion properties and could improve the antimicrobial activity and drugs/agents. However, more efficient DDSs are required due to the high prevalence of oral infectious diseases. In this regard, the therapeutic applications of DDSs might be further improved through the development of multifunctional DDSs (e.g., theranostic nanoparticles and stimuli-responsive DDSs) and an enhancement in their efficacy. The multifunctional DDSs enable researchers to monitor the response to the treatment and control the release behavior of the loaded drugs. Also, the results from the literature can be used to improve the properties of the DDSs (e.g., toxicity and stability). These results can further help to minimize the cost of the treatment by reducing the drug dose.

## Figures and Tables

**Table 1:** Various drug carriers used for the treatment of oral infectious diseases.

Carrier	Therapeutic Agent	Oral Infectious Diseases	Results
Chitosan nanoparticles	Glass ionomer cement and titanium oxide nanoparticles	Dental caries	Chitosan nanoparticles incorporating glass ionomer cement and titanium oxide nanoparticles enhanced the antimicrobial (*Streptococcus mutans* (*S. mutans*)) activity by approximately 1.7-fold [35].
Mesoporous silica nanoparticles (MSNs)	Chlorhexidine	Dental caries	Chlorhexidine-loaded MSNs demonstrated antibacterial activity against biofilms of *S. mutans* (minimum inhibitory concentration (MIC): 100 µg/mL), *Streptococcus sobrinus* (MIC: 200 µg/mL), *Fusobacterium nucleatum* (*F. nucleatum*) (MIC: 100 µg/mL), *Aggregatibacter actinomycetemcomitans* (MIC: 100 µg/mL), and *Enterococcus faecalis* (MIC: 200 µg/mL) [36].
Micelle nanoparticles	Triclosan	Dental caries	Triclosan-loaded micelle nanoparticles inhibited the initial biofilm growth of *S. mutans* by 6-log colony-forming unit (CFU)/hydroxyapatite (HA) disc compared to the untreated control [37].
Liposomes nanoparticles	0WHistatin 5 peptide	Oral candidiasis	Liposomes could increase the cytotoxicity effects of 0Whistatin 5 by approximately 13-fold against *Candida albicans* (*C. albicans*) [38].
PEG-PLGA nanoparticles	Dodonaea viscosa var. Angustifolia (DVA)	Dental caries	DVA-loaded PEG-PLGA nanoparticles demonstrated antibacterial activity against biofilms of *S. mutans* by 8-fold compared to the blank nanoparticles [39].
Hydrogel	Tinidazole	Periodontitis	Tinidazole-loaded hydrogel (monomethoxy poly(ethylene glycol)-block-poly(d,l-lactide) (mPEG-PDLLA)) could increase the t_1/2_ (h), t_max_ (h), and area under the curve (AUC)_0–168_ (h µg/mL) of tinidazole by 16-, 8-, and 21-fold, compared to the control group, in a rabbit periodontitis model [40].
Hydrogel	Histatin-5	Oral candidiasis	Histatin-5-loaded hydrogel (hydroxypropyl methylcellulose (HPMC)) could increase the antifungal (*C. albicans*) effects of histatin-5 by approximately 9-fold compared to the control hydrogel [41].
Dendrimer	Apigenin	Dental caries	The apigenin-loaded dendrimer demonstrated an increase in the antibacterial (*S. mutans*) activity by 1.6-fold compared to the control nanoparticle [42].
PLGA/chitosan composite microsphere	KSL-W peptide	Periodontitis	KSL-W-loaded PLGA/chitosan composite microsphere, compared to the control group, demonstrates significant antibacterial (*F. nucleatum*) activity (inhibition zone of 0 and 2.26 cm in control and KSL-W-loaded PLGA/chitosan composite microsphere receiving groups, respectively) [43].

## Data Availability

Not applicable.
